# Effect of luting materials and root third on glass fiber posts bonding strength after hydrothermal aging

**DOI:** 10.1371/journal.pone.0339002

**Published:** 2026-01-23

**Authors:** Suellen Tayenne Pedrosa Pinto, Carlos Rangel de Moura Oliveira, Mário Tanomaru-Filho, José Maurício dos Santos Nunes Reis

**Affiliations:** 1 Department of Dental Materials and Prosthodontics, School of Dentistry, Sao Paulo State University - UNESP, Araraquara, SP, Brazil; 2 Private Practice, Sao Paulo, SP, Brazil; Indiana University School of Dentistry, UNITED STATES OF AMERICA

## Abstract

**Statement of the problem:**

Teeth with extensive coronal destruction face the challenge of retaining the restorative materials; however, glass fiber posts cemented with resin materials can help to address the issue. The retention of these posts depends on the bond strength to the dentin structure and may vary significantly along the root thirds.

**Purpose:**

This study aimed to evaluate the effect of different luting materials and root thirds on the bonding strength of glass fiber posts to radicular dentin after hydrothermal aging.

**Materials and methods:**

Seventy bovine incisors were divided into 7 groups (n = 10), according to the luting materials: Panavia V5 (*PV5*), Rebilda DC (*RDC*), LuxaCore Z (*LCZ*), Allcem Core (*ACC*), RelyX Ultimate (*RXU*), RelyX U200 (*RXU200*) or Fuji Plus (*FP*). After posts cementation, the roots were thermocycled (10,000 cycles; 5–55°C), sectioned into thirds, and tested for push-out bond strength (2.0 kN; 0.5 mm/min). Failure patterns were classified using a stereomicroscope, according to the material and root third. Data were statistically analyzed (α = 0.05).

**Results:**

Bond strength significantly differed among root thirds for *PV5*, *ACC*, *RXU*, and *RXU200* (p < 0.05), while no significant differences were found for *RDC*, *LCZ*, and *FP* (p ≥ 0.05). *PV5* and *RXU* exhibited the highest overall bond strength values. *PV5* presented means of 14.97 ± 3.68 MPa (cervical), 11.08 ± 3.12 MPa (middle), and 10.47 ± 2.45 MPa (apical), while *RXU* showed 16.97 ± 4.22 MPa, 16.48 ± 3.61 MPa, and 6.51 ± 1.26 MPa, respectively. Overall, *PV5* and *RXU* demonstrated superior and more consistent bond strength across root thirds, whereas the other cements displayed lower and more heterogeneous results.

**Conclusions:**

The luting material influenced the bond strength of glass fiber posts to intraradicular dentin. Dual-cure resin cements with self-etch adhesive systems that contain functional monomers, such as *PV5* and *RXU*, achieved more predictable adhesion, particularly in deep and complex regions.

**Clinical implications:**

Analysis of bond strength and failure patterns supports clinical decision-making by identifying luting cements with superior retention, even in challenging regions, contributing to greater treatment longevity.

## Introduction

Teeth with extensive coronal structure loss — resulting from deep caries, large restorations, fractures, or non-conservative treatments — often require endodontic therapy, sometimes with a prosthetic purpose. In such cases, the lack of sufficient remaining tooth structure compromises the retention and support of the coronal restoration, making the restorative prognosis more challenging [1–3]. In this context, the use of intraradicular posts is often necessary [2,3]. Glass fiber posts are commonly used because of their Young’s modulus (30–50 GPa) that is closer to that of dentin (18.6 GPa), which favors stress distribution [4–6]. These types of posts are cemented with resin or resin-modified materials and depend on the adhesion of the cements to the intraradicular dentin [7–10]. This adhesion, in turn, depends on the quality of the two interfaces that make it up and that are directly connected [11,12].

These two adhesive interfaces can be individually described and analyzed. The first is defined as the bond between the surface of the glass fiber posts and the luting material. The second is the adhesion between the intraradicular dentin and the luting material. There are many studies based on elements that can influence the longevity of adhesion [8,12–16]. It is worth highlighting the simplified post-and-core systems, as they are able to optimize clinical time by integrating the process of cementing the intraradicular post and the construction of the filling core into a single step, resulting in the formation of a “monoblock”. This approach eliminates the need for the conventional two-step protocol, in which the intraradicular post is first cemented, and the coronal core is subsequently built [7,17]. Although these systems reduce the number of adhesive interfaces, they may contribute to polymerization-shrinkage stresses inside the canal, especially due to the high inorganic filler content of these materials [7,18]. Considering the wide range of materials and techniques available for restoring teeth with extensive coronal destruction, it is important to understand the bond strength between the substrates involved and the factors that may influence it [10,19,20]. One of the methods accepted and recommended in the literature to mechanically analyze the adhesion of the dentin-cement-post complex is the push-out bond-strength test, whose acceptance is due to its practical and reliable methodology and its ability to evaluate different root thirds [18,21–23]. Therefore, it is essential to examine the limitations of different adhesive systems and luting materials used in restorations associated with intraradicular posts, considering adhesive challenges such as inadequate dentin retention, poor post adaptation, and polymerization-shrinkage stress.

Restoring endodontically treated teeth with extensive coronal structure loss presents clinical challenges that may compromise adhesion, particularly in deep regions such as the apical third. Proper control of intraradicular dentin hybridization, including moisture management after acid etching and removal of adhesive excess, is essential to achieving reliable bonding. Therefore, the aim of this study was to evaluate the bond strength and failure patterns of different luting systems used in restorations associated to glass fiber posts. The investigation focused on the bond strength of the materials in the cervical, middle, and apical thirds of the root after hydrothermal aging. The null hypothesis was that there would be no significant difference in bond strength among the luting systems across the different root thirds.

## Materials and methods

This study was approved by the Animal Research Ethics Committee under local institutional protocol, registered under number 13/2019. The teeth were obtained through donation from slaughterhouse Terra do Boi, located in Caçu, Goiás, Brazil. As an establishment under state inspection (SIE No. 1459/14), the slaughterhouse follows strict sanitary control protocols, with oversight by a specialized technical team, ensuring animal welfare and the quality of by-products derived from slaughter. This regulation guarantees that the animals are free from clinical signs of disease and that the donated tissues pose no risk of contamination by toxic substances or infectious agents. After donation, the teeth were stored in 0.1% thymol solution for 24 h to ensure initial disinfection, in accordance with standard protocols for the preservation of biological samples [4,9,18]. Considering standardization criteria, seventy extracted single-rooted bovine teeth were selected. All specimens were intact, with a root length of at least 20–21 mm, no curvature, fractures, cracks, resorptions, dilacerations, or other root defects, and with complete root development. Measurements of the roots in the buccolingual and mesiodistal directions were made along the different thirds of the root (coronal, middle, and apical) using a digital caliper (DIN 862, Mitutoyo, Miyazaki, Japan), to ensure that standardized teeth dimensions were selected. Digital periapical radiographs were taken in the same directions in order to measure the intraradicular width, through readings in the ImageJ software version 1.51 (U.S. National Institutes of Health, Bethesda, Maryland, United States of America). The values for the width of the root canals were determined from the drill dimensions of the glass fiber posts (Whitepost DC1; FGM Produtos Odontológicos, Joinville, SC, Brazil) [4,18].

The coronal portion of all teeth was removed by transversal cut using a diamond saw under copious water irrigation (Isomet 2000; Buehler Ltd., Lake Buff, IL, United States of America), always adopting a 19 mm height for the remaining root from cervical to apical. Afterwards, the root canals were instrumented using the Bassi Logic rotary system (Easy, Belo Horizonte, MG, Brazil) up to a 40 file with 0.05 taper, under irrigation with 5.0 mL of sodium hypochlorite at 2.5% (Asfer, São Caetano do Sul, SP, Brazil), preserving 1.0 mm apical during instrumentation. Afterwards, EDTA 17% (Biodynamics, Ibiporã, PR, Brazil) was inserted into the canal for 60 s, followed by irrigation with 10 mL of saline solution. The canal was filled using the single cone technique with gutta-percha and AH plus endodontic cement (Dentsply Maillefer, Ballaigues, Switzerland). The endodontic access was sealed with resin modified glass ionomer material (Vitremer; 3M ESPE, St. Paul, MN, United States of America). Then, the roots were stored in artificial saliva for 7 days [4,24,25].

Subsequently, desobturation was initiated with a heated Rhein instrument, followed by Gates Glidden #2 and 3 and #2–4 Largo drills (Dentsply-Maillefer Instruments SA, Ballaigues, Switzerland), preserving a quantity of 5.0 mm of apical obturation. To remove the remaining filling material, an ultrasonic tip (E4D; Helse Ultrasonic, Santa Rosa de Viterbo, SP, Brazil) was used, activated by the device Ultrawave XS (Ultradent Products Inc, South Jordan, United States of America) at a frequency of 30 kHz and power of 25%, on the canal walls. This procedure was performed with the aid of a surgical microscope (MC-M1232, DF Vasconcellos, Valença, RJ, Brazil), with a 13 × magnification. Afterwards, all teeth were radiographed again in order to verify cleanliness and to certify the ideal intraradicular operation. Then, each root canal was prepared with the WhitePost DC1 drill (FGM Products Odontologist, Joinville, SC, Brazil) to adapt it to the glass fiber posts dimensions and taper [6,25].

Prior to cementation, the WhitePost DC 1 glass fiber posts (FGM Produtos Odontológicos, Joinville, SC, Brazil) were cleaned with 99% isopropyl alcohol and received a layer of silane (FGM Produtos Odontológicos, Joinville, SC, Brazil) for 60 s, followed by compressed air drying. Then, the roots were randomly distributed into 7 experimental groups (n = 10), according to the luting system to be used (Table 1) for the cementation of the glass fiber post. Randomization was performed using a simple draw method. For this, each specimen was sequentially numbered from 1 to 70 and then randomly assigned, one by one, to the 7 experimental groups (n = 10) in a rotating manner. This intercalated allocation process avoided block assignment and ensured balanced and unbiased distribution across groups. The radicular dentin of the teeth was treated according to the manufacturers’ directions of each luting material (Table 2).

**Table 1:** Material/manufacturer, code, cure mode, classification, adhesive systems and basic compositions of adhesive and cement materials.

Material/ Manufacturer	Code	Cure	Classification	Adhesive system	Adhesive composition*	Cement composition*
Panavia V5 (Kuraray, New York, NY, USA)	*PV5*	Dual	Adhesive resin cement	Tooth Primer (Kuraray), Dual polymerization, Self-etching	10-MDP, HEMA, hydrophilic aliphatic dimethacrylate, catalysts, water.	Bis-GMA, TEGDMA, 10-MDP hydrophobic aromatic dimethacrylate, hydrophilic aliphatic dimethacrylate, Initiators, UA-accelerators, silanized barium glass components, silanized fluoroaluminosilicate glass, colloidal silica, silanated aluminum oxide, camphorquinone dl, pigments.
Rebilda DC (Voco, Cuxhaven, Germany)	*RDC*	Dual	Post-and-core flowable composite	Futurabond U (Voco), Dual polymerization, Self-etching	Bis-GMA, UDMA, DDDMA. BHT, dibenzoyl, peroxide, CQ, silica, barium borosilicate glass ceramic, UA-Accelerators.	Bis-GMA, TEGDMA, hydrophobic aromatic dimethacrylate, hydrophilic aliphatic dimethacrylate, initiators, accelerators, silanized barium glass filler, silanized fluoroaluminosilicate glass filler, colloidal silica, silanized aluminum oxide, camphorquinone, pigments.
Luxacore Z dual (DMG, Hamburg, Germany)	*LCZ*	Dual	Post-and-core flowable composite	Luxabond (DMG), Dual polymerization, Total Etch	Barium glass particles, fumed colloidal silica, nanocomposite and zirconium dioxide (50% volume) and bis-GMA (50% volume).	Barium glass, fumed colloidal silica, zirconium dioxide nanocomposite in a matrix of dental resins based on bisphenol A-glycidyl methacrylate.
Allcem Core (FGM, Joinville, SC, Brazil)	*ACC*	Dual	Post-and-core flowable composite	Amber Universal (FGM), Light curing, Universal adhesive	Methacrylate monomers (TEGDMA, BIS-GMA, 10-MDP) camphorquinone, co- initiators, barium-aluminum- silicate glass microparticles, silica dioxide nanoparticles, inorganic pigments and preservatives.	Bis-GMA, Bis-EMA, TEGDMA, photoinitiator, co-initiator, catalysts and pigments.
RelyX Ultimate (3M ESPE, St. Paul, MN, USA)	*RXU*	Dual	Adhesive resin cement	Single Bond Universal (3M ESPE), Dual polymerization, Universal adhesive	BIS-GMA, HEMA, 10-MDP, silica treated with silicon, ethyl alcohol, decamethylene dimethacrylate, water, acrylic and itaconic acid copolymer, camphorquinone, N,N-dimethyl-p-toluidine, 2-dimethylaminoethyl methacrylate, methyl ethyl ketone.	Glass powder surface-modified with methacrylic acid, 2-methyl-3-(trimethoxysilyl)propyl ester, and phenyltrimethoxysilane, hydroxymethyl, dimethacrylate, phosphorus oxide, triethylene glycol dimethacrylate (TEGDMA), silane-treated silica, sodium persulfate, and trimethylhexanoyl peroxide.
RelyX U200 (3M ESPE, St. Paul, MN, USA)	*RXU200*	Dual	Self-adhesive resin cement	Not applicable.	Not applicable.	Glass powder, surface modified with 2- propenoic acid, 2 methyl-.3-(trimethoxysilyl)propyl ester, bulk material, phenyltrimethoxy silane, substituted dimethacrylate, 1,12-dodecane dimethycrylate, 2,4,6(1H,3H,5H)-Pyrimidinetrione, 5-phenyl-1- (phenylmethyl)-, calcium salt (2:1),silane treated silica, 2-Propenoic acid, 2-methyl-, [(3- methoxypropyl)imino]di-2,1-ethanediyl ester, calcium hydroxide, sodium p-toluenesulfinate, titanium dioxide, 2-propenoic acid, 2-methyl-, 1,1’-[1-(hydroxymethyl)-1,2-ethanediyl] ester, reaction products with 2-hydroxy-1,3-propanediyl dimethacrylate and phosphorus oxide, TEGMA, oxide glass chemicals, sodium persulfate, tert-butyl peroxy-3,5,5-trimethylhexanoate, acetic acid, copper(2+) salt, monohydrate.
Fuji Plus (GC America, Aslip, IL, USA)	*FP*	Self-curing	Resin-modified glass ionomer cement	Not applicable.	Not applicable.	Powder: 95% aluminofluorsilicate glass and 5.0% polyacrylic acid powder. Liquid: 50% distilled water, 40% polyacrylic acid and 10% carboxylic acid.

* Information given by the respective manufacturers.

**Table 2:** Luting procedures performed according to each proposed material.

Material	Luting procedure
**Panavia V5**	**Tooth conditioning:** Application of the Tooth Primer self-etching adhesive system for 20 s with the aid of a microbrush. Adhesive excesses removed with absorbent paper and light air jet for 5.0 s. **Cementation:** To apply the cement, the material was extruded from the automix syringe until a homogeneous mixture was obtained, discarding the first 5.0 mm. It was applied inside the entire canal through an intraradicular tip and to the post surface using the same tip. The glass fiber post was manually positioned in the root canal with light vibrations and rotation movements and the cervical cement excesses were removed with a microbrush. **Light-curing:** The polymerization was carried-out using a VALO device (Ultradent Products Inc, South Jordan, USA), at a power of 1000 mW/cm^2^, for 40 s on the occlusal surface of the root.
**Rebilda DC**	**Tooth conditioning:** Application of the FuturaBond U self-etching adhesive system for 20 s with the aid of a microbrush. Adhesive excesses removed with absorbent paper and light air jet for 5.0 s. **Cementation:** To apply the cement, the material was extruded from the automix syringe until a homogeneous mixture was obtained, discarding the first 5.0 mm. It was applied inside the entire canal through an intraradicular tip and to the post surface using the same tip. The glass fiber post was manually positioned in the root canal with light vibrations and rotation movements and the cervical cement excesses were removed with a microbrush. **Light-curing:** The polymerization was carried out using a VALO device (Ultradent Products Inc, South Jordan, USA), at a power of 1000 mW/cm^2^, for 40 s on the occlusal surface of the root.
**LuxaCore Z**	**Tooth conditioning:** Etching the root canal with 35% phosphoric acid (DMG Etching Gel) for 15 s. After washing for 30 s and removing the excess with absorbent paper and light air jets for 5.0 s, a drop of “Pre-Bond” was applied for 15 s inside the root canal, excesses removed with absorbent paper and air jet for 5.0 s. The excess was removed with an absorbent paper cone and then a drop of “Bond A” was mixed with a drop of “Bond B” for 5.0 s and actively applied for 20 s. **Cementation:** To apply the cement, the material was extruded from the automix syringe until a homogeneous mixture was obtained, discarding the first 5.0 mm. It was applied inside the entire canal through an intraradicular tip and to the post surface using the same tip. The glass fiber post was manually positioned in the root canal with light vibrations and rotation movements and the cervical cement excesses were removed with a microbrush. **Light-curing:** The polymerization was carried out using a VALO device (Ultradent Products Inc, South Jordan, USA), at a power of 1000 mW/cm^2^, for 40 s on the occlusal surface of the root.
**Allcem Core**	**Tooth conditioning:** Etching the root canal with 35% Ultra-Etch phosphoric acid (Ultradent Products Inc, South Jordan, USA) for 15 s. The root canal was washed for 30 s and dried with absorbent paper and light air jets for 5.0 s. Two drops of the Ambar Universal adhesive system were applied inside the channel for 10 s each, with subsequent removal of excess with absorbent paper and air jet for 5.0 s, and light jets of air for 10 s. The photoactivation of the adhesive was carried out for 20 s with the VALO device. **Cementation:** To apply the cement, the material was extruded from the automix syringe until a homogeneous mixture was obtained, discarding the first 5.0 mm. It was applied inside the entire canal through an intraradicular tip and to the post surface using the same tip. The glass fiber post was manually positioned in the root canal with light vibrations and rotation movements and the cervical cement excesses were removed with a microbrush. **Light-curing:** The polymerization was carried out using a VALO device (Ultradent Products Inc, South Jordan, USA), at a power of 1000 mW/cm^2^, for 40 s on the occlusal surface of the root.
**RelyX Ultimate**	**Tooth conditioning:** Etching the root canal with 35% Ultra-Etch phosphoric acid (Ultradent Products Inc, South Jordan, USA) for 15 s. The root canal was washed for 30 s and dried with absorbent paper and light air jets for 5.0 s. A layer of Single Bond Universal was actively applied for 20 s and the excess was removed with absorbent paper and light air jets for 5.0 s. **Cementation:** The pastes (base and catalyst) were dispensed in a paper block and mixed for 20 s. The cement was applied to the root canal and to the post surface using the applicator tip of a Centrix syringe (DFL, Rio de Janeiro, Brazil), being the first 5.0 mm dispensed. It was applied inside the entire canal through an intraradicular tip. The glass fiber post was manually positioned in the root canal with light vibrations and rotation movements and the cervical cement excesses were removed with a microbrush. **Light-curing:** The polymerization was carried out using a VALO device (Ultradent Products Inc, South Jordan, USA), at a power of 1000 mW/cm^2^, for 40 s on the occlusal surface of the root.
**RelyX U200**	**Tooth conditioning:** No prior conditioning (self-adhesive material). **Cementation:** The pastes (base and catalyst) were dispensed in a paper block and mixed for 20 s. The cement was applied to the root canal and to the post surface using the applicator tip of a Centrix syringe (DFL, Rio de Janeiro, Brazil), being the first 5.0 mm dispensed. It was applied inside the entire canal through an intraradicular tip. The glass fiber post was manually positioned in the root canal with light vibrations and rotation movements and the cervical cement excesses were removed with a microbrush. **Light-curing:** The polymerization was carried out using a VALO device (Ultradent Products Inc, South Jordan, USA), at a power of 1000 mW/cm^2^, for 40 s on the occlusal surface of the root.
**Fuji Plus**	**Tooth conditioning:** Conditioning of root canal dentin for 10 s with polyacrylic acid (DFL, Rio de Janeiro, Brazil), followed by vigorous washing for 30 s, removal of excess with absorbent paper points, and light air jets for 5.0 s. **Cementation**: Two shallow scoops of powder and two drops of liquid were provided, and the cement was mixed for 30 s. The cement was applied to the root canal and to the post surface using the applicator tip of a Centrix syringe (DFL, Rio de Janeiro, Brazil), and the post was seated with slight vibrations and rotary movements. Cervical excesses were removed with a microbrush. **Light-curing:** Not required. The post was held in position for 10 min to allow for complete chemical polymerization of the cement.

After cementation of the posts, the entire sample (N = 70) was subjected to thermocycling: 10.000 cycles with immersion baths in distilled water at temperatures of 5–55ºC ± 2.0ºC; lasting 30 s for each temperature, with a 5.0 s delay between immersions in the different tanks. Subsequently, the roots were embedded in polystyrene-based resin (Maxi Rubber, Diadema, SP, Brazil) in 15 x 15 mm PVC cylinders, positioned vertically with the help of a dental surveyor (BioArt B2, São Carlos, SP, Brazil). The roots were sectioned in the apical, middle, and cervical thirds with thicknesses of 2.0 mm, respectively, from 1.0, 4.0, and 7.0 mm from the cervical to the apical region. The sectioned specimens were then polished using 1200 and 1500 grit silicon carbide paper (Norton, São Paulo, SP, Brazil) under running water to remove surface scratches and irregularities. Subsequently, the samples were cleaned with a soft brush and air jets to eliminate any debris prior to testing [26–28].

The push-out mechanical test was performed for each of the sections obtained using an electromechanical testing machine (EMIC DL 2000, São José dos Pinhais, PR, Brazil), with a 2.0 kN load cell, operating at constant speed of 0.5 mm/min. According to the root thirds, different metallic cylinders were used for complete extrusion of the glass fiber posts. The diameters of the cylinders were 1.3, 0.9 and 0.5 mm for the cervical, middle and apical thirds, respectively [26,28,29]. In all sections, the apical face was positioned to come into contact with the metallic cylinder of the push-out device, in such a way that the applied force was always applied from apical to cervical, providing adequate extrusion of the posts.

The maximum bond strength was measured by displacing the glass fiber post from the root canal. The maximum force to displace the glass fiber posts was obtained in Newton (N) and converted into megapascal (MPa) [26,28]. Before performing out the bond strength test, the root diameters of the top and bottom surfaces of all the specimens were measured under 20 × magnification using a stereomicroscope (Leica Microsystems-M80, Wentzler, Germany). Then, the adhesive area (mm^2^) was calculated for conversion of N values into MPa. The formula used was: π ⋅ (R+r) ⋅ [h^2^+(R-r)^2^]^0.5^, where “R” and “r” represent, respectively, the radius of the top and bottom portions and “h” represents the thickness of each slice in millimeters. The values obtained in N for each mechanical test were divided by the adhesive area values of each specimen.

The bond strength data (MPa) were submitted to normality (Shapiro-Wilk; p ≥ 0.05) and homogeneity of variance (Levene; p ≥ 0.05) tests. The bond strength produced in the cervical, middle and apical thirds (n = 10) was statistically analyzed for each material separately, in order to compare the intra-group results in the different root thirds. In addition, separate analyzes of the root thirds (cervical, middle or apical third) were performed in order to compare the materials with each other. For this, one-way analyses of variance were used (α = 0.05), considering *Material*, with the results of the different root thirds or *Root third*, with the results of the different materials for each region as isolated factors. Tukey’s HSD post-hoc tests were used (α = 0.05) when a significant effect was verified. For the analyses, the SPSS version 24.0 program (Statistical Package for Statistical Science Inc., Chicago, IL, United States of America) was used.

The failure pattern was analyzed by a single calibrated operator using 40 × magnification in a stereomicroscope (Leica Microsystems-M80, Wentzler, Germany). The failure patterns were organized and tabulated, according to the materials and root thirds. Percentage calculations were then carried out to determine the incidence of each type of failure for each experimental condition. Scanning electron microscopy (SEM) analysis was then carried out using a JSM 5600LV microscope (JEOL Ltd, Rio de Janeiro, RJ, Brazil), operating at 15 kV, with 40 × magnification, to obtain representative images of each failure mode. The failure modes were classified according to Leandrin et al. [4] and Ramos et al. [30] as type 1 (adhesive failure between luting material and dentin), type 2 (adhesive failure between luting material and glass fiber posts), type 3 failure (cement cohesive) and type 4 (mixed failure, when adhesive and cohesive failures occur concomitantly). However, in this study, cohesive failures of the glass fiber posts were also observed, being classified as type 5 [4,23,30].

## Results

One-way ANOVAs revealed statistically significant differences (p < 0.05) in bond strength among the materials across each root third (Table 3; horizontal comparisons). When the materials were evaluated separately, statistically significant differences (p < 0.05) were found only among the root thirds of *PV5*, *ACC*, *RXU*, and *RXU200* (Table 3; vertical comparisons).

**Table 3:** Means and standard deviations (±) of bond strength values (MPa) for the different materials within each root third and for each material among the root thirds, along with the results of Tukey’s HSD post-hoc test.

	*PV5*	*RDC*	*LCZ*	*ACC*	*RXU*	*RXU200*	*FP*
**Cervical Third**	14.97 ± 3.68 A a	6.68 ± 1.96 B	8.14 ± 3.89 B	8.98 ± 3.45 B a	16.97 ± 4.22 A a	6.43 ± 2.33 B a	6.69 ± 2.53 B
**Middle Third**	11.08 ± 3.12 B ab	6.55 ± 2.38 CD	8.62 ± 2.60 BC	5.03 ± 1.89 CD b	16.48 ± 3.61 A a	4.61 ± 0.60 D ab	7.05 ± 2.08 CD
**Apical Third**	10.47 ± 2.45 A b	4.69 ± 1.60 CD	8.36 ± 2.74 AB	4.93 ± 1.73 CD b	6.51 ± 1.26 BC b	3.05 ± 1.15 D b	5.61 ± 2.09 BCD

Horizontally, equal capital letters denote statistically similar results to each other (p ≥ 0.05). Vertically, equal lowercase letters denote statistically similar results to each other (p ≥ 0.05).

Comparing the materials, *PV5* and *RXU* produced the highest bond strength in the cervical root third (p < 0.05). For the middle and apical thirds, the findings were more heterogeneous among the materials.

When the root thirds were compared, all materials showed a similar pattern, with significantly higher bond strength in the cervical third compared with the apical third (p < 0.05), and statistically similar values between the middle and apical thirds (p ≥ 0.05), except for *RXU*, which produced lower results in the apical third compared with the middle third (p < 0.05). No significant differences (p ≥ 0.05) were observed for *PV5*, *RXU*, and *RXU200* between the cervical and middles thirds.

**Fig 1:**
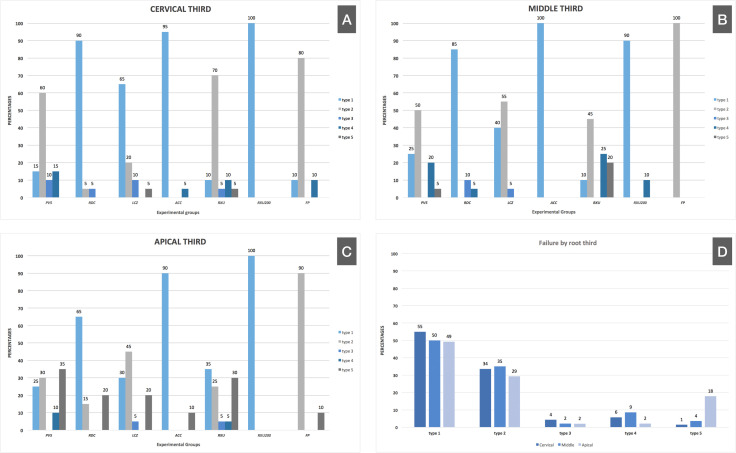
Incidence of failure for each experimental condition within each root third (A-C), and overall failure by root third (D).

**Fig 2:**
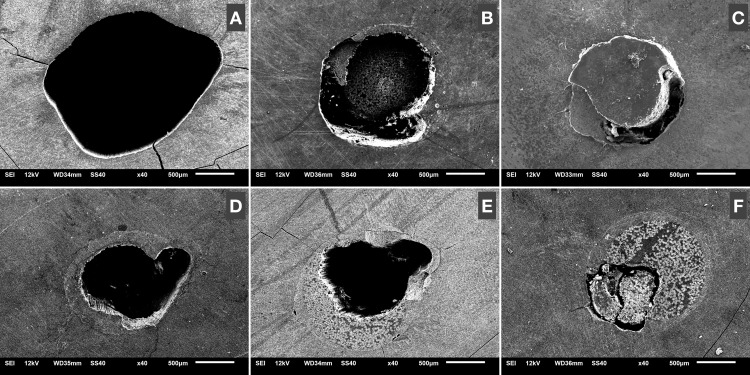
Representative SEM images of the types of failures. A: type 1; B: type 2; C and D: type 3; E: type 4; and F: type 5. All images were taken using a scanning microscope at 40 × magnification.

After the push-out mechanical strength test, a qualitative analysis of the specimens was carried out. The data were tabulated and analyzed by percentages, and the results were presented graphically (Fig 1) to show the frequency of failure patterns for each experimental condition and root third. Scanning electron microscopy (SEM) analysis provided representative images of each failure pattern, as shown in Fig 2. Images C and D show the apical and cervical thirds with cement remnants, respectively, indicating the presence of cohesive failure of the cement.

## Discussion

The bond strength of glass fiber posts to radicular dentin in different thirds by means of using the luting materials *PV5*, *RDC*, *LCZ*, *ACC*, *RXU*, *RXU200* and *FP*, after hydrothermal aging, varied significantly, rejecting the inferred null hypothesis.

In order to reduce the bias of the samples, the intraradicular preparation for the glass fiber posts was standardized, so that they were juxtaposed to the walls of the root canal. The adequate fit of the posts ensures better mechanical imbrication and smaller cementation thickness, reducing the incidence of voids and cracks, as well as reduces the stress of polymerization shrinkage, which predisposes to bonding failures [1,7,31]. Grandini et al. [13] report that the greater the thickness of the luting agent, the greater the number of voids and, consequently, the greater the fragility of the adhesion; aspect corroborated by several authors [16,19,29]. The analysis of the bond strength through the mechanical push-out test favors the analysis by root thirds in a more simplified and efficient way. This mechanical test generates transversal force with homogeneous distribution causing parallel failures in the adhesive interface (dentin-cement-post), with a lower incidence of premature failures [8,19].

The absolute numerical difference in the values of intraradicular bond strength between the root thirds was notorious for most of the luting materials evaluated, which generally behaved in a decreasing manner, from cervical to apical. This corroborates other studies [15,16] that studied intraradicular cementation and adhesion, attributing the different results between the cervical and apical thirds to factors such as luting agent selection, sensitivity of the adhesive technique, dentinal morphological difference, anatomical aspects, difficulty to access the apical third during the adhesive procedures such as cleaning, bonding agent application and light curing. The apical third has a lower density of dentinal tubules and a smaller amount of intertubular dentin, which reduces bond strength to adhesive and luting systems [19,32]. The technical sensitivity of intraradicular dentin hybridization directly influences the bonding efficiency. Among the various adhesive systems, the total-etch has superior technical sensitivity, both in terms of acid etching time, as well as washing quality and substrate moisture control [33]. Despite this, from the statistical observations, little variation was observed between the middle and apical thirds, with the exception of the *RXU* adhesive resin material. Still, there were no statistically significant differences, regardless of the root third, between the *FP* resin modified glass ionomer cement and *RDC* and *LCZ* post-and-core materials, contrary to the findings of other studies [15,16]. These results are likely related to methodological differences, particularly the standardized intraradicular preparation protocol adopted in this study, which aimed to ensure the closest possible adaptation between the fiber post and the root canal walls. Proper juxtaposition of the post promotes better mechanical interlocking and a thinner cement line, reducing void formation, adhesive failures, and polymerization shrinkage stress, all of which directly impact bonding performance [16,18,19].

Luting materials can be classified according to their chemical composition, curing mode (chemical, dual or light curing), interaction with the dental substrate (chemical, mechanical or micromechanical) and application mechanism/interaction with various substrates (adhesive, self-adhesive). Materials for cementing glass fiber posts should preferably be dual-curing or chemically curing, especially due to the difficulty of light penetration in the apical region of the root [10]. Resin-modified glass ionomer cements have good adhesion to the dentin substrate because they have an intrinsic chemical bond that interacts with dentin calcium, as well as a mechanical bond and adhesion due to the resin monomers incorporated into their composition. Resin cements may have functionalized monomers in their composition to promote chemical bonding to the dentin substrate and to the glass fiber posts [10,34]. Thus, intraradicular bond strength results can be higher when compared to total-etch adhesive systems and conventional resin cements. When comparing the luting agents in relation to the evaluated root thirds, we observed that the *PV5* adhesive resin cement, whose application system consists of the use of a self-etching adhesive system with dual polymerization, produced outstanding performance regardless of the evaluated root thirds.

*RXU* and *PV5* exhibited high bond strength values, particularly in the cervical and middle thirds. This performance can be attributed to the presence of the functional monomer 10-MDP, which is contained in the *PV5* cement and in the adhesive system used with *RXU*. Chemically, 10-MDP establishes stable ionic bonds with calcium in hydroxyapatite, forming durable MDP-Ca salts [9,35]. Mechanically, demineralized dentin allows monomer infiltration, promoting hybrid layer formation and resin tag development, which together produce micromechanical interlocking and strengthen the adhesion interface [9,35]. In addition, effective copolymerization between the resin cement and the adhesive system may have contributed to favorable mechanical behavior across the root thirds. *RXU* demonstrated superior values in the cervical and middle thirds, locations where clinical control of dentin moisture and adhesive application is more predictable [12,16,19]. However, the increased technique sensitivity and anatomical constraints of the apical third may limit adhesive performance, which is consistent with previous findings [18,20]. *PV5* demonstrated high bond strength along the entire canal, including the apical third. This result may be associated with its self-etch adhesive system and dual-curing mechanism, which reduce technique sensitivity by eliminating prior phosphoric acid etching. In addition, the incorporation of 10-MDP in its formulation likely contributed to stable bonding throughout the root length [8,9,35,36]. The Ambar Universal adhesive system of the *ACC* material, despite having 10-MDP in its composition, is light-curing; a fact that may have directly influenced the results of this material, especially in the middle and apical thirds, even though it was used in the total-etch technique, due to the difficulty for the adequate photopolymerization of the bonding agent in the apical area. It was not the scope of this study to compare the viscosity and filler content of different luting agents, but the post-and-core materials have a higher filler concentration, providing greater viscosity [7]. *ACC* is a resin material that is included in this classification and, according to the study of Barreto et al. [2], also has higher viscosity, due to its higher filler concentration in comparison to the self-adhesive resin cement *RXU200*. Thus, despite the greater adhesion expected to the *ACC* material, as it has 10-MDP as a component of its Ambar Universal adhesive system, it produced similar bond strength to the self-adhesive resin cement *RXU200*. In the current investigation, etching with phosphoric acid was used prior to the application of Ambar Universal adhesive system and cementation with *ACC* material, unlike the study by Barreto et al. [2], which used this bonding agent in the self-etching protocol. However, there was similarity between the results of these previously cited authors and the present study. It is necessary to highlight that there is a scarcity of studies with the *ACC* material, limiting the search for justifications despite the found results.

When comparing the bond strength results of the different root thirds for each material, *RDC*, *LCZ* and *FP* did not produce statistically significant differences among the root thirds. The homogeneity results among the different root thirds presented by the *LCZ* can be justified by the use of its self-cure activator in combination with its bonding agent, capable of maintaining the bond strength at deeper root thirds, regardless of the limited light conditions in the apical area [37].

The resin-modified glass-ionomer luting material *FP* did not show high numerical values of bond strength when compared to some resin cements, such as *PV5*, regardless of the root third, or *RXU*, in the cervical and middle thirds, but showed comparable results to *ACC*, *RDC*, *RXU200* and *LCZ* in the cervical and middle thirds, and to *ACC*, *RDC*, *RXU200*, *LCZ* and *RXU* in the apical third. The homogeneity of the results obtained along the root thirds may be due to its composition, which favors chemical adhesion, in addition to its chemical cure, not being influenced by the adhesive technique, as well as light conditions for photopolymerization. According to Cury et al. [38], during its curing process, the resin-modified glass-ionomer cement initially consumes all the water in its composition. This initial reaction causes material shrinkage; however, as curing progresses, a hygroscopic expansion occurs — an increase in volume due to the absorption of additional moisture from the dentinal tubules [39,40]. This expansion provides a better bond strength with dentin along the entire length of the root canal [33]; a fact that justifies the homogeneous results produced by FP [39,40].

The *RDC* material also showed homogeneity among the root thirds. Similar results were previously reported [17], suggesting that its self-etching adhesive system (Futurabond U) and the degree of resin cement conversion may be related to such findings. The self-etching adhesive system can interact with dentin in two ways, micromechanically and chemically. The micromechanical interaction occurs due to the polymerization of monomers that infiltrate the dentin, and this system does not completely remove the smear layer as it has a medium aggressive pH. Therefore, it causes partial dentinal demineralization, exposing a network of collagen fibers that will be infiltrated by the monomers. On the other hand, chemical adhesion occurs through ionic bonds between conventional adhesive monomers and the residual calcium of hydroxyapatite. In addition, this post-and-core system presents lower technical application sensitivity. Another extremely important factor is that this material is a dual-curing composite, which compensates the lower light penetration in the apical third and favors its degree of conversion along the entire length of the canal [17]. Considering the post-and-core materials *RDC* and *LCZ* are compared, it can be observed that both produced uniform adhesion in all root thirds. On the other hand, *LCZ* produced high bond strength results in the apical third, fact that might be related to the radicular dentin pre-preparation protocol recommended by its manufacturer, in which phosphoric acid etching is used.

Self-adhesive resin cements are known for their low technique sensitivity and ease of handling and clinical application. When comparing *RXU200* and *RXU*, both manufactured by the same company, it becomes clear that their intended clinical applications differ. The *RXU* adhesive resin cement requires prior dentin conditioning, which facilitates hybrid layer formation and results in improved bond strength, as confirmed by the findings of this study [6,8,18]. In contrast, the *RXU200* self-adhesive resin cement aims to simplify the technique by eliminating the etching step. Because it does not promote smear layer removal or deep dentin demineralization, *RXU200* does not form a true hybrid layer or resin tags. Its acidic monomers interact superficially with the hard tissues, mainly through the smear layer. This interaction is less effective due to the limited demineralization capability caused by a rapid local pH increase. These characteristics likely explain the lower bond strength values observed for *RXU200*, especially in the apical third, where anatomical and clinical challenges such as limited access and moisture control further compromise adhesive performance [6,8,9,18,19].

The results observed regarding failure patterns showed that the highest number of failures occurred at the intraradicular cement-dentin adhesive interface. This analysis is relevant as it helps identify the region of greatest susceptibility to failure within the bonding interfaces. Factors such as the complexity of the radicular dentin substrate, with significant morphological variations from the cervical to the apical third, including lower dentinal tubule density, especially in the middle and apical thirds, hinder the formation of the hybrid layer and resin tags. In other words, they compromise the infiltration of adhesive monomers between collagen fibrils, promoting adhesive failures in this region [8,12,16,19].

Additionally, the stress generated by polymerization shrinkage of the cement may concentrate at the cement-dentin interface, especially when there is an uneven thickness of the cement line [16,19]. Although a rigorous intraradicular preparation protocol was adopted in this study to ensure a thin and standardized cement layer, the bond strength results indicate that cement distribution within the canal was not entirely uniform. This is evidenced by the differences observed among the values recorded for each root third, suggesting a likely thickening in the apical region, which consistently showed the lowest bond strength values across all groups. Greater polymerization shrinkage in these deeper regions may generate increased stress on the dentinal walls, compromising the adhesion at the cement-dentin interface and contributing to the higher incidence of adhesive failures in this third, predominantly failures at the dentin-cement interface [18,19]. The findings of Amiri et al.[19] support these observations, indicating that such conditions can significantly compromise bond strength in the apical region. In contrast, the cement-post interface showed lower susceptibility to failure, possibly due to the greater chemical compatibility between the resin-based components of the cement and the resin matrix of the glass fiber post, along with the homogeneous surface of the post, which facilitates micromechanical adhesion along its entire length [41]. These findings are in line with previous studies [18,21,42–44] that identify the dentin-cement interface as the most vulnerable point in glass fiber post adhesive cementation.

The present study has limitations such as the analysis of material viscosity and penetrability and its role in intraradicular adhesion. Additional and longitudinal studies evaluating the influence of factors such as cementation line thickness, histological and anatomical variations should be conducted to elucidate the direct correlation of these bond strength results with the success and prognosis of restorative therapy in endodontically treated teeth with extensive coronal destruction.

## Conclusions

The choice of luting material system significantly influences the bond strength of glass fiber posts to intraradicular dentin, especially in challenging regions such as the apical third. In clinical scenarios requiring effective adhesion in deep and complex root canals, the use of dual-cure self-etch adhesive and resin cement systems containing functional monomers such as 10-MDP (e.g., *PV5* and *RXU*) may provide more predictable bonding outcomes. In contrast, etch-and-rinse systems such as *LCZ* or light-dependent adhesive systems like that of *ACC* exhibited greater technique sensitivity and lower performance in difficult-to-access areas. Moreover, the resin-modified glass-ionomer cement *FP* demonstrated consistent performance across root thirds, suggesting it may be a viable option when simplified protocols and reduced technique sensitivity are desired. Therefore, the selection of luting materials should consider not only ease of application but also the adhesive strategy and anatomical challenges of each root third to optimize retention and restoration longevity.

## Supporting information

S1 Data Raw data repository. (XLSX)
